# Barriers to hospital and tuberculosis programme collaboration in China: context matters

**DOI:** 10.3402/gha.v8.27067

**Published:** 2015-09-24

**Authors:** Guanyang Zou, Rebecca King, John Walley, Jia Yin, Qiang Sun, Xiaolin Wei

**Affiliations:** 1 China Programme, COMDIS Health Services Delivery Research Consortium, University of Leeds, Shenzhen, China; 2 Institute for International Health and Development, Queen Margaret University, Edinburgh, UK; 3 Nuffield Centre for International Health and Development, University of Leeds, Leeds, UK; 4 Devision of Health System, Policy and Management, School of Public Health and Primary Care, Chinese University of Hong Kong, Hong Kong, China; 5 Centre for Health Policy and Management, School of Public Health, Shandong University, Jinan, China

**Keywords:** tuberculosis programme, general hospitals, collaboration, open systems

## Abstract

**Background:**

In many developing countries, programmes for ‘diseases of social importance’, such as tuberculosis (TB), have traditionally been organised as vertical services. In most of China, general hospitals are required to report and refer suspected TB cases to the TB programme for standardised diagnosis and treatment. General hospitals are the major contacts of health services for the TB patients. Despite the implementation of public–public/private mix, directly observed treatment, short-course, TB reporting and referral still remain a challenge.

**Objective:**

This study aims to identify barriers to the collaboration between the TB programme and general hospitals in China.

**Design:**

This is a qualitative study conducted in two purposefully selected counties in China: one in Zhejiang, a more affluent eastern province, and another in Guangxi, a poorer southwest province. Sixteen in-depth interviews were conducted and triangulated with document review and field notes. An open systems perspective, which views organisations as social systems, was adopted.

**Results:**

The most perceived problem appeared to be untimely reporting and referral associated with non-standardised prescriptions and hospitalisation by the general hospitals. These problems could be due to the financial incentives of the general hospitals, poor supervision from the TB programme to general hospitals, and lack of technical support from the TB programme to the general hospitals. However, contextual factors, such as different funding natures of different organisations, the prevalent medical and relationship cultures, and limited TB funding, could constrain the processes of collaboration between the TB programme and the general hospitals.

**Conclusions:**

The challenges in the TB programme and general hospital collaboration are rooted in the context. Improving collaboration should reduce the potential mistrust of the two organisations by aligning their interests, improving training, and improving supervision of TB control in the hospitals. In particular, effective regulatory mechanisms are crucial to alleviate the negative impact of the contextual factors and ensure smooth collaboration.

Tuberculosis (TB) is a global public health problem with 8.7 million TB patients and 1.4 million deaths in 2011. More than 95% of TB deaths occur in low- and middle-income countries (1). Global TB control adopts a systematic public health approach called directly observed treatment, short-course (DOTS) as recommended by the World Health Organization (WHO) since the 1990s. DOTS is largely implemented by public sector services under national TB programmes (NTPs). However, many patients seek care from a variety of health services that are not formally included within the DOTS framework (2). Hospitals remain a challenge for providing TB services. A survey in seven larger African and Asian countries showed that TB treatment in hospitals was often associated with non-referral or non-reporting to NTPs, poor adherence to the standard NTP regimen, lack of patient-tracing mechanisms, or unknown treatment outcomes (3). As a component of the health system, TB control has to engage all providers in the health system to achieve the targets of case detection and treatment success. This takes many forms in different countries: public–private mix, private–private mix, and public–public mix (PPM-DOTS) (4). In China, this includes the cooperation between the public hospitals and the TB programme (public–public mix) and the involvement of village doctors (public–private) in TB control.

China has the second largest TB burden in the world, with one million new TB cases each year (1). China's TB control is led by the health bureau, relying on four levels of TB programmes: national, provincial, prefectural, and county. The TB programme is normally hosted within Centres for Disease Control and Prevention (CDC) at all levels. The TB programme at the county level is the endpoint of TB control. At the county level, the TB programme collaborates with a three-tier general health services network combining county hospitals, township hospitals, and village clinics (5). The health bureau, as the government authority, oversees such collaboration. Currently, the most popular form of PPM-DOTS is called ‘TB programme-based model’. In most of China, the TB programme has its own TB clinic, which provides standardised diagnosis and treatment for general TB cases, either self-reported to the TB clinic or referred from general health services, including general hospitals. The TB programme also supervises reporting and referrals of TB suspects and cases and traces all the referred cases who do not visit the TB programme within 3 days. The general hospital has a limited role in treating TB, except for treating complicated and severe TB cases. General TB cases treated in the TB programme enjoy free care, and costs of essential anti-TB drugs, X-rays, and sputum checks are covered by the TB programme.

Before 2003, TB control in China was inadequate with the case-detection rate stagnating at around 30% (6). TB was supposed to be diagnosed and treated in the TB programme. However, the national survey showed that 91% of the symptomatic TB patients who visited health facilities used general health services as their first contact, including 34% for the general hospitals. Only 13% of the patients diagnosed by the general hospitals were referred to the TB program (7). Public hospitals who treated TB patients rarely used DOTS regimens and nor did they report TB to the TB programme or provide free diagnosis and drugs to TB patients. Moreover, public hospitals often over-prescribed drugs and examinations for profit (8, 9). Many studies have reported the long shopping cost and diagnosis delays of TB patients seeking care among public hospitals before their diagnosis at the TB programme (10–12).

In 2005, China achieved the WHO targets for TB control with 80% of new smear-positive TB case detection (6), largely thanks to the post-SARS public health systems strengthening. Since 2003, the national government has increased funding for public health institutions, revised law on the control of infectious diseases, and established the world's largest internet-based communicable-disease reporting system (6). In 2004 and 2005, the Ministry of Health issued two notices: ‘Notice about Further Strengthening of TB Report and Patient Management’ and ‘Operational Methods to Refer and Trace TB Cases’, respectively. The standard of TB reporting, referring, and notification from general hospitals was further developed, and the mechanism of collaboration between general hospital and TB programme was re-established. Strengthening the collaboration between public hospitals and the TB programme also received support from international agencies such as the Global Fund.

Thanks to PPM-DOTS and post-SARS public health strengthening, reporting and referral rates have greatly improved in recent years (13–15). General hospitals remain one of the most important contacts for TB patients. However, one study showed that more than 20% of TB suspects and patients needing referral from hospitals did not reach TB programmes (13). Our studies found that half of the general TB patients (without comorbidities) chose general hospitals as their first contact with health care (16, 17). Before being referred to the TB programme, 80% of these patients visited the general hospitals and nearly half of them were hospitalised in the general hospitals, largely contributing to the catastrophic health expenditures of the TB patients (16, 17).

In recent years, a new form of PPM-DOTS, known as ‘designated hospital-based model’, has emerged in some provinces of China, where a ‘designated’ hospital provides the standard TB diagnosis and treatment, while the TB programme remains the basic management unit. However, this model of collaboration did not necessarily reduce patients’ out-of-pocket payments (16, 18, 19). Our qualitative study also suggested that the recent reform has met with great health system challenges, especially in western areas (5). By the end of 2012, less than 30% of the counties had established the ‘designated hospital-based model’ (20). Currently, the TB programme-based model remains the most common model of PPM-DOTS. Very few qualitative studies have specifically focused on the collaboration between the TB programme and general hospitals in this model. Also, none have adopted a systems perspective that helps to understand the configuration of factors related to context, process, and output of such collaboration. Using an open systems perspective, this study aims to identify the barriers to collaboration between the TB programme and general hospitals in China.

## Methods

### Conceptual framework

Inter-organisational collaboration can be viewed as an open system, which is defined as a coalition of shifting interest groups, strongly influenced by environmental factors, who develop goals by negotiating structure, activities, and outcomes (21). Open systems theory argues that organisations are social systems made up of a structuring of events or processes, attitudes, beliefs, and motivations of humans. The theory stresses the complexity and variability of parts, the looseness of connections, amorphous system boundaries, and attention to process, not structure (21).

**Fig. 1:**
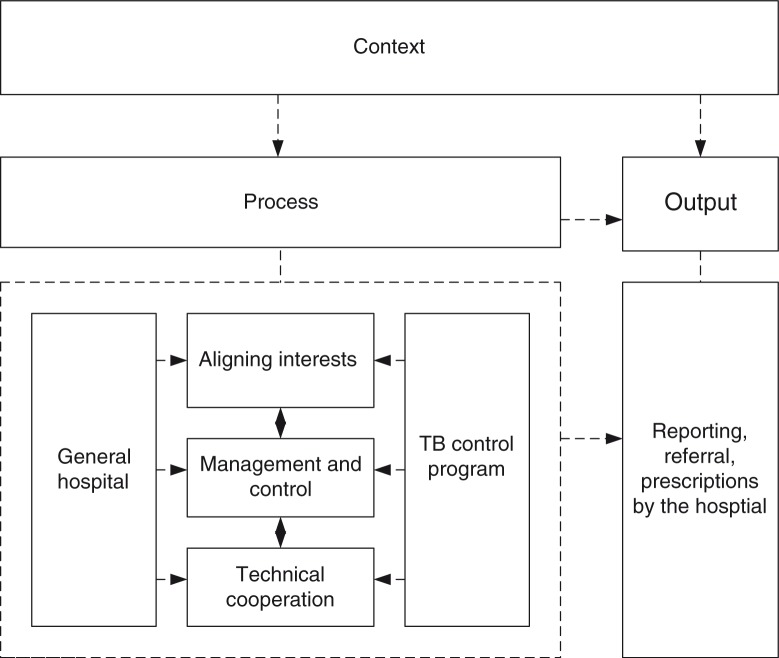
An open systems framework to study TB programme and general hospital collaboration.

Health services and organisations, such as the TB programme and general hospitals, are highly specialised and fragmented. They are often characterised by differing rules, boundaries, funding streams, and institutional and professional cultures. Inter-organisational collaboration allows organisations to constructively explore their differences and address barriers beyond their own limited visions of what is possible. They do this by transforming organisational inputs, such as financial, regulatory, and technical resources, as facilitated or constrained by the context (22). The processes of inter-organisational collaboration could involve addressing three major sub-systems: 1) the maintenance sub-system, which aligns different interests and values to hold the social structure together by reducing variability; 2) the management sub-system, which improves collaboration efficiency through control, coordination, directing, regulatory mechanisms, and authority structure; and 3) the technical sub-system, which provides technical support to achieve the essential goals of the collaboration (23–26). An open systems framework is adapted to help to understand the collaboration of the TB programme and general hospitals (Fig. 1).

### Study design

This study is part of a larger project that explored the different models of the TB programme and general hospital collaboration in China (5, 16, 18, 19). A qualitative study design was adopted, which was particularly valuable when seeking to explore implementation processes in depth (27). This study was conducted in two counties: one in an eastern province in China and the other in a south-western province. It is always of interest to understand policy implementation issues across eastern and western China. ‘East’ and ‘west’ are two geographical areas with political significance in China. Eastern areas are normally better off whereas the western areas tend to be poorer. The prevalence of TB in western areas is twice as high as that of eastern areas (28). Zhejiang is a more affluent province on the eastern coast, and Guangxi (located in the southwest of China, bordering Laos and Vietnam) is poorer. ‘ZD’ is the relatively rich county site from the eastern province, and ‘GP’ is the relatively poor county site from the western province. (The research sites are abbreviated to protect the anonymity of the respondents.) ZD has significantly higher TB notification rates than GP. Both sites reported having achieved more than an 85% cure rate for new smear-positive cases and a 70% case-detection rate. Both sites have similar TB service delivery systems. The models of TB programme and hospital collaboration are identical – a ‘TB programme-based model’ in both sites, whereby the general hospitals refer TB suspects and general TB patients to the TB programme for standard diagnosis and treatment.

### Data collection

Sixteen in-depth interviews were conducted in the two sites using semi-structured interview topic guides. The methods and procedures of the in-depth interviews were similar to those used in our published study (5). Purposeful sampling was used, in order to select information-rich cases for in-depth study (29). The potential interviewees were identified based on their relevance to the questions, resulting in eight interviews being conducted in each site (Table 1). In each site, interviewees included leaders and staff from the county health bureau, county general hospital, and county CDC (which hosted the TB programme). The health bureau director was selected because that position led the TB control work, provided funding, and managed the TB control work. The TB programme director and staff were at the core of policy implementation as they treated TB and managed and supervised the hospital reporting and referral. The hospital director played a leading role in policy implementation within the hospital. Doctors working in the outpatient (e.g. respiratory), radiological, and laboratory departments remained crucial in reporting and referring TB suspects and cases. Interviewing inpatient doctors helped to understand their admission behaviour. Public health doctors were key to policy implementation as they monitored and supervised reporting and referrals and served as the focal point for the coordination between the hospital and TB programme. This composition of interviewees reflected the ‘maximum variability principle’, providing rich information from different experiences and perspectives. A general interview guide approach was adopted, allowing the basic topics to be covered and adapted in each interview (30). Topics were loosely structured around key issues related to general hospital and TB programme collaboration, such as governance, funding, communications, and linkages between the TB programme and general hospitals.

**Table 1:** Sampling for the in-depth interviews

Organisation	Positions	ZD	GP
Health bureau	Vice director	1	1
CDC (TB programme)	TB programme director	1	1
	TB programme staff	1	1
General hospital	Director	1	1
	Outpatient doctor	1	1
	Inpatient doctor	1	1
	Radiology staff	1	1
	Public health staff	1	1
Total		8	8

The research team included TB researchers from the University of Leeds based in China and Shandong University. The researchers were experienced in conducting qualitative research in TB and health systems. Their identity as university researchers, instead of health officials, enabled the interviewees to comment on sensitive issues more openly. The county TB programme was responsible for the coordination of the project and communicated with the relevant departments falling into the interview categories. Health workers, available for 1-h talks, were invited for interviews. They were reassured of the research nature and their anonymity, were provided with the research outline, and were asked to sign an informed-consent form before the interview. All interviews were audiotaped and lasted between 40 and 100 min (60 min average). The recording was transcribed by trained postgraduate students from Shandong University and checked by the primary interviewer. Ambiguous or inaudible sections of text were clarified by interviewees via emails or phone calls.

To triangulate the in-depth interview data, we reviewed published and unpublished documents regarding PPM leadership, health financing, financial reports, routine TB reports, and other relevant ‘grey’ literature. During our field work, we also observed TB control activities, and notes were taken. Ethical approval was granted by the Ethical Committee of the School of Public Health of Shandong University.

### Data analysis

Analyses focused specifically on identifying content related to the context, process, and output of TB programme and hospital collaboration. The methods and procedures of qualitative analysis were similar to another published study (5). Specifically, a thematic approach was used (27) to allow for the application of the existing framework (open systems framework) and the inclusion of emerging themes from the data. Analysis was supported by Weft QDA, a computer-assisted qualitative analysis programme. A framework table was progressively established and structured following the reading through of the topic guides and transcripts. Transcripts of each interview were coded into the related themes and sub-themes. The themes and sub-themes were modified, and emerging themes were included following the coding process (Table 2). A team approach was used, which provided a form of researchers’ triangulation (31, 32). In addition, triangulation was performed to refute or confirm emerging findings within each data set across different sources of data. For example, as financial interests began to emerge as a key issue within the interview data, this was also explored within documents and field notes. Triangulation provided a validation process, thereby increasing the construct validity and trustworthiness of our findings. Bearing in mind the potential for ‘leading’ questions, the team also reviewed the questions and participants’ responses, which generally provided balanced and open accounts.

**Table 2:** Example of coding list

Conceptual dimensions	codes
Output	Adherence to TB guidelines Drug prescriptions Hospitalisation
Process	Mistrust between TB programme and hospital Social benefits Financial interests Supervision Technical support
Context	Funding for hospital versus CDC/TB programme TB control system structure Hospital versus CDC resources Medical culture Relationship culture Funding on TB control

## Results

**Fig. 2:**
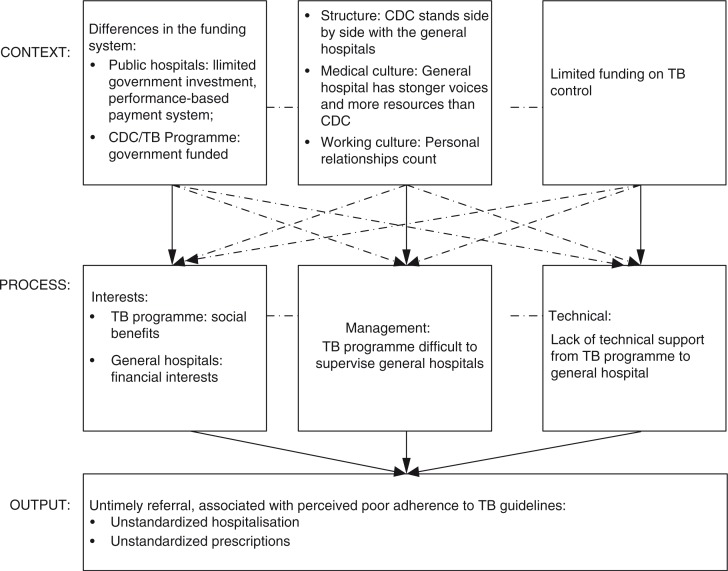
Dynamics of TB programme and general hospital collaboration.

Our study identified barriers to a closer collaboration between TB programmes and general hospitals in a semi-vertical TB control system. These mainly centred on contextual influences on the interests of general hospitals and TB programmes; management of hospital and TB programme collaboration; and technical support from TB programmes to general hospitals; and the perceived output of collaboration, that is, untimely referral, associated with perceived poor adherence to TB guidelines. The two sites presented more similarities than differences. Figure 2 illustrates the dynamics of contextual factors that influence the TB programme and general hospital collaboration.

### Contextual influence on the interests of general hospitals and TB programmes

Salaries and operational costs of TB programmes were mainly funded by the government. However, government investment only accounted for a small amount of fiscal income in the public hospitals. More than 85% of revenue of general hospitals came from selling medications and medical services. It was suggested that differences in the funding system may have influenced the different interests of TB control between these two types of health facilities. It appeared that the TB programme wanted all patients to be treated under the DOTS programme for social benefits, whereas general hospitals tended to retain TB patients for financial interests.

There are conflicts in the interests of TB control between the TB programme and hospital staff anyway. The TB programme has a work target and hopes to detect more patients. But the general hospital wants to make more money. (GP health bureau staff)

The TB programme staff accused general hospitals of only treating TB patients for profits. In general hospitals, a performance-based payment system was implemented that incentivised doctors based on the services they provided and the examinations and drugs they prescribed. As a result, hospitals tend to retain TB patients for profits because referring patients may affect their income. However, the hospital staff disagreed with the criticism from the health bureau and TB programme that they always admitted patients for profit reasons. They maintained they referred TB patients to the TB programme and admitted patients based on their conditions.

Very often we refer the TB suspects and patients once we detect. As we have limited hospital beds, we try to avoid admitting patients. (ZD hospital staff)

Once we detect TB patients, we would explain to them how TB could be treated and what conditions we have here. We told them it was free and professional in the TB programme and recommended them being examined and treated there. We did recommend the serious patients to be hospitalised here. For example, we would admit those elderly people with breathing difficulty directly. (GP hospital staff)

### Contextual influence on the management of hospital and TB programme collaboration

A leadership committee was established at the county level to solve strategic issues of TB control. It included the vice governor and directors of the health bureau and Finance Bureau. Another committee was established at the health bureau level that included the director of the health bureau and directors of general hospitals, the TB programme, and township hospitals. The role of this committee was to solve operational issues such as TB treatment and referrals. However, responsibility for the daily management of TB control remained with TB programmes. TB programmes are responsible for supervising TB tasks in the general hospitals and reporting the results to the health bureau for hospital evaluation. However, TB programme staff often felt helpless in supervising the TB control work by the general hospitals. They reflected that it was difficult to coordinate their relationship with the general hospital or monitor the clinical behaviour of the hospital staff. They even attributed this challenge to the ‘mutual choices’ of both doctors and patients.

If the patients need to be hospitalised, the hospital should report to the TB programme, but they may not do so. We do not have special measures to control the hospitalisation rate of the TB patients in the general hospital. This totally depends on the willingness of the general hospitals and the patients themselves. Patients themselves wouldn't agree to be hospitalised without money. We have no choice. (GP TB programme staff)

The CDC stands side by side with the general hospitals; both are under the leadership of the health bureau. This structure has weakened the TB programme's supervision of the TB control work by the general hospitals. The general hospital seemed to have stronger voices in the health sector as it had more resources generated from health insurances and patients than the CDC which only had a limited number of TB patients. Hospitals are very influential and closer to people's everyday life and so are more well-known than the public health facilities. Sometimes, the director of a general hospital is also the vice director of the health bureau, indicating the importance of medical work in the hospital.

Just like you and me, we can't give orders to each other. The TB programme doesn't have authority over the hospital. The TB programme is helpless if the hospital director does not follow its advice. The director of [the] general hospital is the vice director of health bureau. If the hospital does not refer TB patients, the TB programme has no way of persuading him …. (GP health bureau)

They [general hospitals] are the ‘big brother’ in the health sector, so how can we manage and supervise them? (ZD TB programme staff)

The health bureau should have played an important role in coordinating the relationships between the TB programme and the hospitals. However, this was not necessarily the case. Instead, the personal relationships were regarded as more effective in daily coordination than the regulations or punishments from the health bureau. A small referral incentive was provided to the hospital doctors, whereas those who did not refer or report would face a fine. However, the punishment was rarely practised. For example, general hospitals that were found not referring TB suspects were only informally ‘criticised’ by the health bureau and not necessarily ‘punished’.

Leaders of the health bureau are aware of this, but they could do little about it. Even they are helpless in managing an effective relationship between us [hospital and TB programme]. (ZD TB programme staff)

Certainly, we can punish the hospital, but we can't do that for the political reasons …. The personal relationships between the two organisations [hospital and TB programme] are so important. Just one call can solve problems, if both have a good relationship. (GP health bureau staff)

### Contextual influence on the technical support from the TB programme to general hospital

The TB programme and general hospitals normally met on a monthly basis for meetings or supervisions. The TB programme had the responsibility of training the public health doctors from general hospitals, township hospitals, and village clinics once a year on TB control. The public health work appeared to be less important within the hospital compared with the clinical work because it did not bring major income but, rather, consumed the hospital's budget. Technical support was relatively weak from the TB programme to the general hospitals for TB control. The doctors from internal or respiratory medicine at the general hospitals reported that they had not received any training from the TB programme or other institutions in recent years. However, they did report training, albeit limited, provided by the public health doctors within the hospital, although the quality of this training was a concern.

I don't know if there is any training course. I have never participated for these two years. I just remember the public health doctors within our hospital explained the ‘referral’ notes to us. It was in a mess …. We always forgot which sheet should be given to patients and which sheet [should] be sent back to the public health department. (GP hospital staff)

I have not participated in any training related to TB control in the recent two years. Normally, only one staff can participate in the training each time as we are very busy with our work. (ZD hospital staff)

Lack of training could be associated with limited funding on TB control, which was normally sourced from the local government, the central transfer budget, and external project funding. In general, the study at both sites revealed that the TB control budget was not sufficiently in line with the current TB epidemic and workload. Without enough funding in place, it would be difficult for the TB programme to provide effective support to the general hospitals. Together with lack of supervision and income-generating activities of the hospital, this would compromise the quality of reporting and referral.

The operational cost keeps increasing by year, but the financial input from the government is beyond our control. The budget is fixed and cannot be changed, but we do feel it is not enough. (ZD TB programme staff)

### Untimely referral, associated with perceived poor adherence to TB guidelines

As a result, untimely referral was identified as the biggest barrier for the hospitals and TB programme collaboration. Associated with this problem was the perceivably poor adherence to TB guidelines as reflected by non-standardised prescriptions and hospitalisations.

There was a concern that too many non-standardised hospitalisations occurred in the general hospitals. According to the TB guidelines, TB should be reported to the TB programme once they were diagnosed in the hospital. However, reporting and referring TB patients were often made after the patient had been discharged.

The general hospital would recommend TB patients to be hospitalised first and over half of them were hospitalised. (ZD TB programme staff)

The tendency for over-hospitalisation was concerning due to the potential consequence of increasing the financial burden of TB patients.

Patients are squeezed until the last pence of their money before being referred to us. The patient expenditure in the hospital is very high as the hospital implements the performance-based salary, which is related to the volumes of prescribed drugs and examinations. (ZD TB programme staff)

On the other hand, associated with the TB treatment in the hospital was the non-standardised prescription of TB drugs, which had the potential to cause the development of multidrug-resistant (MDR) TB.

Prescriptions in the hospital are not always standardised. For example, they do not use the quadruple drug (as prescribed by the WHO and NTP); rather, the three-combined drugs without rifampin …. There were also irrational prescriptions of levofloxacin …. One patient was hospitalised for more than 10 days; given intravenous drips of streptomycin. (ZD TB programme staff)

## Discussion

Using an open systems framework, our study identified barriers to collaboration between the TB programmes and the general hospitals. The most perceived problem appeared to be untimely reporting and referral associated with non-standardised prescriptions and hospitalisation by the general hospitals. These problems could be due to financial incentives of the general hospitals, poor supervision from TB programmes to general hospitals, and lack of technical support from TB programmes to general hospitals. However, contextual factors, such as different funding natures of different organisations, prevalent medical and relationship cultures, and limited TB funding, could constrain the processes of collaboration between TB programmes and general hospitals.

Unlike another study that evaluated the integration of clinical TB services in the general hospitals (5), we found more similarities than differences across the richer east and poorer west sites. However, the fact that few thematic differences were observed between two qualitatively different sites does not mean that our results could be applied across the entire health care system in China. The study approach has a limitation in generalisability. However, it provides an in-depth platform from which to apply the open systems theory, which helps in understanding the configuration of context, process, and output of hospital and TB programme collaboration in the context of PPM-DOTS. Although representativeness and sample size are not the major concern in qualitative research (33, 34), future research could be built on these findings with a larger sample size and a mixed design to understand the effect of this collaboration.

In general, this study supported the quantitative finding of our previously published study that nearly half of the uncomplicated TB patients who visited the general hospitals received hospitalisation before referral (16, 17). The average hospitalisation was nearly 20 days, causing a significant financial burden on the TB patients and delayed opportunity to receive the standardised treatment in the DOTS facilities (16, 17). Using an open systems perspective, we disclose the ‘black box’ of hospital and TB programme collaboration in association with the perceivably poor collaborative outputs. The open systems framework views hospital and TB programme collaboration as a social entity and helps to address the speciality, fragmentation, and complexity of the interest groups. The collaboration may be influenced by contextual factors, which shape their interests, management, and technical cooperation (processes).

The general health services have long separated from the vertically orientated services such as the TB programme. However, different funding mechanisms between the general hospitals and TB programmes may have shaped different interests. The post-SARS public health strengthening efforts may have largely reduced the financial interests of the public health institutions such as the CDC (6). However, this change did not improve the partially or self-funded status of the hospitals (5). The public hospitals were encouraged to raise income by charging patients and operating like a private entity, due to the limited government funding (35). The TB programme, fully funded by the government, has a strong commitment to TB control as it pursues ‘social benefits’, whereas general hospitals may compromise this with ‘profit-orientated’ service. These contrasting interests may lead to mistrust between these two organisations, thus damaging collaboration (5, 36). The disagreement over the ‘profit-orientated’ hospitalisation, as reflected in this study, might reflect the different understanding of the hospitalisation criteria and lack of communication between the two organisations. The performance-based incentives for health providers, such as reporting and referral incentives, have played an important part in improving referrals (37). However, the minimal financial incentives, either positive or negative, may not be effective enough for the TB referral system. Appropriate financing mechanisms should be developed to motivate the general hospitals to conduct and support public health work; for example, by encouraging referrals in this case (12).

Contextual factors also shaped the authority and power relationship of collaboration. The parallel position between the CDC (which hosts the TB programme) could reduce the authority of the TB programme to supervise the work of general hospitals. However, the superior status of general hospitals to public health facilities, due to the prevalent medical culture and better resources, could worsen the situation. Another study suggested the importance of a powerful intermediary source, such as the health bureau, in mediating the difficult relationship between the TB programme and general hospitals (5). However, this study suggests that the health bureau is embarrassing in hospital management as income generation is important for hospital development. Relationship culture is popular, which may often replace the rigorous implementation of regulations as an alternative (5, 36, 38). In this case, effective inter-organisational collaboration may require more informal contacts and communications between the organisations (22). However, strong regulatory frameworks with effective implementation are indispensable for effective collaboration. The health bureau should make good use of the PPM-DOTS committee as an important governance platform and play a positive role in monitoring and evaluating the clinical behaviour of the hospitals based on the TB and other PPM-DOTS guidelines.

Consistent with another study (12), we found a general lack of training for general hospital staff on DOTS expansion from the TB programme. Lack of funding for TB control could have a direct impact on the delivery of training. This could result in poor reporting and referral and poor management of TB cases, as was often observed in the non-DOTS hospitals, potentially generating a high prevalence of MDR TB (3). Although the financial interests of the general hospitals and ineffective supervision from the TB programme need addressing, it is important to improve the training of general hospital staff on the identification, reporting, and timely referral of potential TB cases to the DOTS facilities.

## Conclusions

The challenges in the TB programme and general hospital collaboration are rooted in the context. Improving their collaboration should reduce the potential mistrust of the two organisations by aligning their interests and improving training and supervision of TB control in the hospitals. In particular, effective regulatory mechanisms are crucial to alleviate the negative impact of the contextual factors and ensure smooth collaboration.
